# Seven Tesla Evidence for Columnar and Rostral–Caudal Organization of the Human Periaqueductal Gray Response in the Absence of Threat: A Working Memory Study

**DOI:** 10.1523/JNEUROSCI.1757-23.2024

**Published:** 2024-06-26

**Authors:** Alexandra K. Fischbach, Ajay B. Satpute, Karen Quigley, Philip A. Kragel, Danlei Chen, Marta Bianciardi, Larry Wald, Tor D. Wager, Ji-Kyung Choi, Jiahe Zhang, Lisa Feldman Barrett, Jordan E. Theriault

**Affiliations:** ^1^Department of Psychology, Northeastern University, Boston, Massachusetts 02115; ^2^Department of Psychology, Emory University, Atlanta, Georgia 30322; ^3^Department of Radiology, Athinoula A. Martinos Center for Biomedical Imaging, Massachusetts General Hospital and Harvard Medical School, Charlestown, Massachusetts 02129; ^4^Department of Psychological and Brain Sciences, Dartmouth College, Hanover, New Hampshire 03755; ^5^Department of Surgery, University of California, San Francisco, California 94143

**Keywords:** brainstem, high resolution, neuroimaging, periaqueductal gray, working memory

## Abstract

The periaqueductal gray (PAG) is a small midbrain structure that surrounds the cerebral aqueduct, regulates brain–body communication, and is often studied for its role in “fight-or-flight” and “freezing” responses to threat. We used ultra-high-field 7 T fMRI to resolve the PAG in humans and distinguish it from the cerebral aqueduct, examining its in vivo function during a working memory task (*N* = 87). Both mild and moderate cognitive demands elicited spatially similar patterns of whole-brain blood oxygenation level-dependent (BOLD) response, and moderate cognitive demand elicited widespread BOLD increases above baseline in the brainstem. Notably, these brainstem increases were not significantly greater than those in the mild demand condition, suggesting that a subthreshold brainstem BOLD increase occurred for mild cognitive demand as well. Subject-specific masks were group aligned to examine PAG response. In PAG, both mild and moderate demands elicited a well-defined response in ventrolateral PAG, a region thought to be functionally related to anticipated painful threat in humans and nonhuman animals—yet, the present task posed only the most minimal (if any) “threat,” with the cognitive tasks used being approximately as challenging as remembering a phone number. These findings suggest that the PAG may play a more general role in visceromotor regulation, even in the absence of threat.

## Significance Statement

The periaqueductal gray (PAG) is thought to control survival-related behavior and is typically studied using experiments that manipulate threat. Others have proposed that the PAG plays a more general role in bodily regulation, but studies examining PAG function outside of threat-based experimental contexts are rare. We used high-resolution fMRI to examine PAG response in humans during a working memory task, which involves minimal threat. Moderate cognitive demands elicited a well-defined response in ventrolateral PAG, a functional subregion thought to coordinate a “freezing” response to threat. A task where threat is minimal elicited a clear fMRI response in one of the most well-known survival circuits in the brain, which suggests the PAG supports a more general function in brain–body coordination.

## Introduction

Historically, the periaqueductal gray (PAG) has been identified as a key brain structure mediating the “fight-or-flight” and “freezing” responses (for review, see Bandler, 1988; Bandler et al., 1991a), and this functional association of the PAG with threat responses continues to be articulated in recent reviews (Mobbs et al., 2015, 2020; Silva and McNaughton, 2019). Direct chemical stimulation of the PAG elicits a diverse range of motor behaviors and physiological responses—including, in cats, immobility, pupil dilation, vocalization (e.g., hissing or howling), alert positions (Bandler and Carrive, 1988), running and jumping (Zhang et al., 1990), and hypertension and tachycardia (Abrahams et al., 1960), all of which are generally understood as part of the “fight-or-flight” and “freezing” responses (Bandler et al., 1991b; Bandler and Depaulis, 1991; Fanselow, 1994). Likewise, in humans, the PAG is most commonly studied for its role in threat (Coker-Appiah et al., 2013; Hermans et al., 2013; Lindner et al., 2015; Weis et al., 2020; Zhou et al., 2021; Wang et al., 2022; Weis et al., 2022). Deep brain stimulation of the human PAG elicits a desire to escape (Amano et al., 1978), feelings of fear and impending death, and autonomic changes including hyperventilation and increased heart rate (Nashold et al., 1969). Brain imaging studies in humans observed that blood oxygenation level-dependent (BOLD) signal intensity increases in the PAG when participants were chased through a virtual maze (i.e., approached by a simulated predator; Mobbs et al., 2007) and when exposed to a learned cue signaling the onset of an unpleasant respiratory restriction (Faull et al., 2016, 2019). The interpretation of the PAG as a primary region for coordinating survival-based responses has been used to unify these many observations under a single coherent function (Fanselow, 1991; Silva et al., 2016), in which the PAG was thought to play a central role in an evolutionarily ancient “fear circuit” (Panksepp, 2011; Silva et al., 2016; LeDoux and Daw, 2018; McNaughton and Corr, 2018; Lu et al., 2022; Vázquez-León et al., 2023).

The hypothesis that the PAG is a center for survival-based responses unifies a great deal of evidence, but not all evidence. For example, the PAG has been observed to play a functional role in bodily processes at moments when threat is absent (for review, see Carrive, 1993), including control of the heart (M. A. Gray et al., 2009; Wager et al., 2009b; Pereira et al., 2010; Hermans et al., 2013; Hohenschurz-Schmidt et al., 2020), blood pressure control (Green et al., 2005), cardiorespiratory coordination (Dampney et al., 2013), bladder control (Blok et al., 1997; Blok and Holstege, 1998), rapid eye movement sleep (J. Lu et al., 2006), body temperature (Zhang et al., 1997; Chen et al., 2002), trigeminovascular contributions to migraine headache (Knight and Goadsby, 2001), communication (e.g., the vocalization of songbirds; Haakenson et al., 2020), mating behavior (e.g., the control of male copulatory behavior in quails; Carere et al., 2007), and feeding behaviors (e.g., the motivational drive to hunt in mice; Mota-Ortiz et al., 2012; Tryon and Mizumori, 2018). Furthermore, the PAG has extensive connectivity with regions across the entire neuraxis, from the spinal cord and the brainstem to the cerebral cortex, placing it at a critical integration point in brain–body communication (for review, see Carrive and Morgan, 2012). Given this evidence, we hypothesized that the well-known involvement of PAG in response to predatory threat is consistent with a more fundamental function: the PAG may coordinate and regulate internal bodily (visceromotor) systems in service of efficient regulation of the body (called allostasis). On this interpretation, threat responses represent only one of many contexts where the autonomic nervous system, the endocrine system, the immune system, and tissues of the body must be coordinated with each other and with motor movements (for review, see Benarroch, 2012; Motta et al., 2017). If this is correct, and the PAG does play a more general regulatory role (rather than a threat-/survival-specific role), then organized patterns of PAG response may be observed in task-based contexts involving effort, but minimal threat.

Critically, well-known columnar functional distinctions within the PAG set some expectations for how its response is organized. Within the PAG, prior work has identified dorsomedial (dmPAG), dorsolateral (dlPAG), lateral (lPAG), and ventrolateral (vlPAG) functional columns (Bandler et al., 1991a; Carrive, 1993). In the context of threat-/survival-based research, dl/lPAG is thought to support active coping “fight-or-flight” responses to predatory threats, whereas dmPAG is thought to support similar responses to aggressive conspecifics, and vlPAG is thought to support passive coping or “freezing” responses to painful threats (Zhang et al., 1990; Morgan et al., 1998; Gross and Canteras, 2012). For example, stimulating dl/lPAG, in rodents and cats, increases cardiac rates and respiratory rates (RRs; Bandler et al., 1991c; Carrive and Bandler, 1991; Depaulis et al., 1992; Zhang et al., 2005; Subramanian et al., 2008) and elicits explosive running and jumping (Fardin et al., 1984; Zhang et al., 1990; Bandler et al., 1991c; Morgan et al., 1998). In contrast, stimulating vlPAG decreases cardiac rates and RRs (Bandler et al., 1991c; Carrive and Bandler, 1991; Depaulis et al., 1992; Lovick, 1992; Subramanian et al., 2008) and induces an absence of movement in cats (Zhang et al., 1990) and “freezing” behavior in rats (Carrive et al., 1997; Morgan et al., 1998). In humans, anticipated breathlessness increases the BOLD signal in vlPAG (Faull et al., 2016, 2019; Tinoco Mendoza et al., 2023), whereas subcutaneous (deep muscle) and cutaneous pain increases the BOLD signal in vlPAG and lPAG, respectively (Tinoco Mendoza et al., 2023). Observations that correspond to this functional localization along PAG columns would increase our confidence in an observed pattern of results, given that these functional distinctions have been strongly established in prior work.

**Figure 1.**
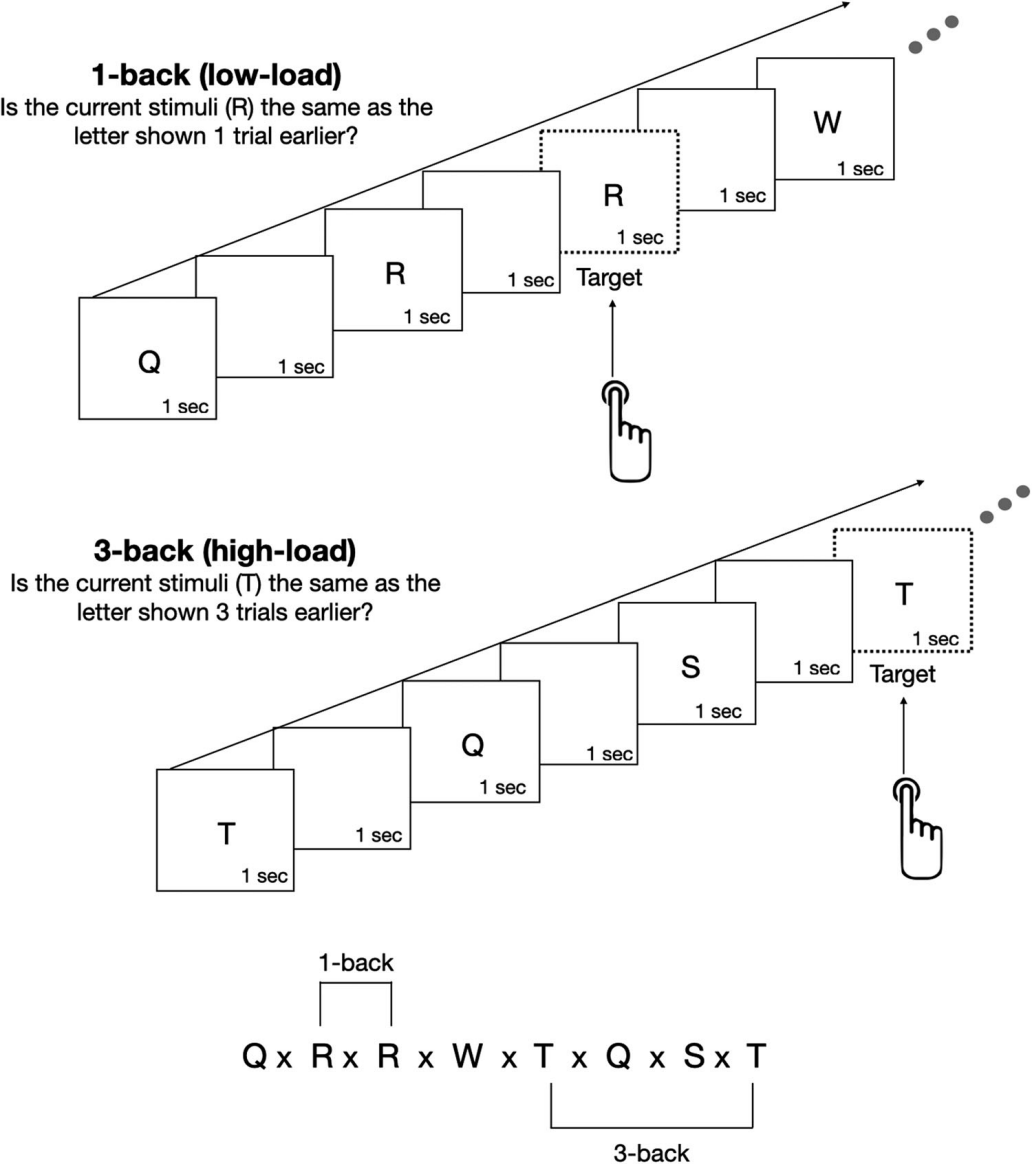
Schematic overview of the working memory N-back task. An example of a 1-back (mild cognitive demand) and 3-back (moderate cognitive demand) task showing a sequence of trials. For every trial, a single letter was presented at the center of the visual field for 1 s. Following the 1 s stimuli presentation, participants were given an additional 1 s to respond before the next trial began. Participants were instructed to respond with a button press if the target stimulus matched the stimulus *n* (either 1 or 3) positions back.

In the present study, we examined changes in PAG activity in the context of a mildly-to-moderately challenging N-back working memory task, which aims to simulate energetic, cognitive, and physiological demands within the range of those typically encountered in daily life. Participants observed a sequence of letters and responded with a key press when a letter was repeated, either from the prior trial (1-back) or three trials prior (3-back; Fig. 1). This task required that participants simultaneously attend to and remember target stimuli while inhibiting or completing task-relevant motor responses. The 1-back task could be conceptually described as easier than remembering a phone number, and so we consider it to fall well outside the traditional survival-based contexts in which PAG function has previously been tested.

Imaging resolution in 1.5 and 3 T MRI scanners is insufficient to resolve small brainstem nuclei, due to both low signal-to-noise ratios (SNRs) and voxel resolution. In contrast, ultra-high resolution 7 T imaging, used in the present work, provides a substantial increase in the SNR (Linnman et al., 2012; Satpute et al., 2013). An earlier report included the first 24 participants from the current dataset (all the participants who were available for analysis at that time) and used univariate and multivariate methods to distinguish PAG BOLD responses to 1-back (mild cognitive demand) and 3-back (moderate cognitive demand) conditions (Kragel et al., 2019). This earlier study reported greater BOLD signal intensity increases in the 3-back (vs the 1-back) condition, which were localized to the right rostral vlPAG. The present work builds on this earlier report, with a sample size (*N* = 87) that affords the power to identify functional distinctions among PAG columns. We also improved our localization procedure, identifying PAG columnar subregions by their radial degree. As in prior work (Kragel et al., 2019), we aligned subject-specific PAG masks to a group template to estimate the voxelwise BOLD response during the N-back task. For PAG columns, tract-tracing techniques in nonhuman animals typically produce symmetrical results (Carrive et al., 1987; Zhang et al., 1990; Carrive and Bandler, 1991; Li et al., 1993; Sandkühler and Herdegen, 1995; Holstege et al., 1997; Jansen et al., 1998; Paredes et al., 2000), and so we expected that PAG activity would be symmetric along the PAG midline. We recorded cardiac interbeat interval (IBI) and RR to examine whether columnar organization supported visceromotor functions and whether this visceromotor support could possibly account for any task-elicited changes observed during the working memory task.

## Materials and Methods

### Participants

One hundred forty participants were recruited from the Greater Boston area and provided informed consent in accordance with guidelines set forth by the Partners’ Healthcare Institutional Review Board. Consented participants completed two scanning sessions and were paid $150 for each scan (i.e., $300 upon study completion). Eighteen participants either withdrew or ended the MRI scan session before starting the working memory task. Nineteen participants were excluded due to poor image quality (e.g., large artifacts compromising analysis), as assessed by visual inspection in Mango version 4.1 (RRID: SCR_009603). Sixteen participants were excluded for excessive head motion [>0.5 mm framewise displacement in >20% of repetition times (TRs) in the run; see below, MRI preprocessing]. Our final sample consisted of 87 participants (*M*_age_* *= 27.0 years; *SD*_age_* *= 6.1 years; 39 female, 47 male, 1 nonresponse). For self-reported ethnicity, 13% of participants identified as Hispanic. For self-reported race, 62% identified as White, 25% identified as Asian, and 9% identified as Black. Of the 87 participants, three did not provide a response for race, and one did not provide a response for ethnicity. The participant sample was generally highly educated (24% had some graduate education, 25% had completed college/university, 37% had some college/university, 10% had completed high school, and 2% had not completed high school). Peripheral physiological recordings were inspected for artifacts (see below, Peripheral physiological recording and quality assessment), and only nonartifactual recordings were included in analyses. Of the 87 participants with usable fMRI data, 59 had artifact-free cardiac data, and 47 had artifact-free respiratory data.

All participants were between 18 and 40 years old, right-handed, fluent English speakers, with normal or corrected to normal vision and no known neurological or psychiatric illnesses. Participants were excluded from participating if they were pregnant, were claustrophobic, or had any metal implants that could cause harm during scanning.

### Experimental paradigm

Participants completed a visual N-back working memory task during fMRI scanning, based on the experimental design of prior studies (Kirchner, 1958; Gevins and Cutillo, 1993; Gray et al., 2003; Wager and Smith, 2003; van Ast et al., 2016). On each trial, a letter (Q, W, R, S, or T) was presented at the center of the visual field for 1 s followed by a blank screen for 1 s (Fig. 1). Participants were instructed to respond with a button press when the letter on the screen matched the letter presented *n* trials ago where *n* was either 1 or 3. The task was administered during a single scanning run, across 12 blocks, each consisting of 10 trials (120 trials total). Each block began with a 3 s cue indicating the current trial type (1-back or 3-back). The 1-back and 3-back blocks were presented in 1-3-3-1 or 3-1-1-3 order (counterbalanced across participants), and each block was followed by 25 s of resting fixation. Stimuli were classified into two categories; a “target” was a stimulus that met the N-back criteria for a match (e.g., N-back either one or three trials ago), and all other stimuli were classified as “nontargets.” The task had fixed proportions of 20% targets and 80% nontargets. Of the nontargets, 12.5% were classified as lure trials—i.e., 2-back matches in the 1-back blocks and 2- or 4-back matches in the 3-back blocks. The proportion of lure trials was the same for both 1-back and 3-back blocks.

The task was administered in MATLAB (MathWorks; RRID:SCR_001622), using the Psychophysics Toolbox extensions (Kleiner, 2007; RRID:SCR_002881). Stimuli were made visible to participants by projecting them onto a mirror attached to the head coil. Responses were recorded using an MR-compatible button box.

To familiarize participants with the task, participants completed a practice N-back task session on a laptop immediately before the scan session. These practice sessions mirrored the task used during scanning, including blocks of 1-back and 3-back trials, for a total of 48 trials.

### MRI data acquisition

Gradient-echo planar imaging (EPI) BOLD-fMRI was performed on a 7 T Siemens MRI scanner at the Athinoula A. Martinos Center for Biomedical Imaging at Massachusetts General Hospital. The scanner was built by Magnex Scientific, with the MRI console, gradient and gradient drivers, and patient table provided by Siemens. Functional images were acquired using the GRAPPA-EPI (generalized autocallibrating partially parallel aquisitions) sequence: echo time = 28 ms; TR = 2.34 s; flip angle = 75°; slice orientation = transversal (axial), anterior to posterior phase encoding; voxel size = 1.1 mm isotropic; gap between slices = 0 mm; number of axial slices = 123; field of view = 205 × 205 mm^2^; GRAPPA acceleration factor = 3; echo spacing = 0.82 ms; bandwidth = 1,414 Hz per pixel; and partial Fourier in the phase encode direction = 7/8. A custom-built 32-channel radiofrequency coil head array was used for reception. Radiofrequency transmission was provided by a detunable bandpass birdcage coil.

Structural images were acquired using a T1-weighted EPI sequence, selected so that functional and structural images had similar spatial distortions, which facilitated coregistration and subsequent normalization of data to the MNI space (Renvall et al., 2016). Structural scan parameters were as follows: echo time = 22 ms; TR = 8.52 s; flip angle = 90°; slice orientation = transversal (axial); voxel size = 1.1 mm isotropic; gap between slices = 0 mm; number of axial slices = 126; field of view = 205 × 205 mm^2^; GRAPPA acceleration factor = 3; echo spacing = 0.82 ms; bandwidth = 1,414 Hz per pixel; and partial Fourier in the phase encode direction = 6/8.

### MRI preprocessing

Functional and structural MRI data were preprocessed using the fmriprep pipeline, version 1.1.2 (Esteban et al., 2019, 2020; RRID: SCR_016216), a Nipype-based tool (Gorgolewski et al., 2011; Esteban et al., 2020; RRID: SCR_002502). Full pipeline details can be found at https://fmriprep.org/en/1.1.2/workflows.html. Spatial normalization of anatomical data to the 2009c ICBM 152 Nonlinear Asymmetrical template (Fonov et al., 2009; RRID: SCR_008796) was performed through nonlinear registration with the antsRegistration tool of ANTs version 2.1.0 (Avants et al., 2008; RRID: SCR_004757), using brain-extracted versions of both T1w (T1-weighted) volume and template. Brain tissue segmentation of the cerebrospinal fluid (CSF), white matter (WM), and gray matter (GM) was performed on the brain-extracted T1w using FAST in FSL version 5.0.9 (Zhang et al., 2001; RRID: SCR_002823). Functional data were slice time corrected using 3dTshift from AFNI version 16.2.07 (Cox, 1996; RRID: SCR_005927) and motion corrected using MCFLIRT (FSL version 5.0.9; Jenkinson et al., 2002). This was followed by coregistration to the corresponding T1w using boundary-based registration (Greve and Fischl, 2009) with nine degrees of freedom, using FLIRT (FMRIB’s Linear Image Registration Tool; FSL version 5.0.9; Jenkinson and Smith, 2001; Jenkinson et al., 2002). Motion-correcting transformations, BOLD-to-T1w transformation, and T1w-to-template (MNI) warp were concatenated and applied in a single step using antsApplyTransforms (ANTs version 2.1.0) using Lanczos interpolation. Physiological noise regressors were extracted using the aCompCor method (Muschelli et al., 2014), taking the top five principal components from subject-specific CSF and WM masks, where the masks were generated by thresholding the WM/CSF masks derived from FAST at 99% probability, constraining the CSF mask to the ventricles (using the ALVIN mask; Kempton et al., 2011), and eroding the WM mask using the binary_erosion function in SciPy version 1.6 (Virtanen et al., 2020). Framewise displacement (Power et al., 2014) was calculated for each functional run using the implementation of Nipype. Many internal operations of fmriprep use Nilearn (Abraham et al., 2014; RRID: SCR_001362), principally within the BOLD-processing workflow. For all participants, the quality of brain masks, tissue segmentation, and MNI registration was visually inspected for errors using the HTML figures provided by the fmriprep pipeline.

### fMRI analysis

To estimate BOLD signal intensity during the working memory N-back task, a general linear model was applied to preprocessed functional time-series (GLM; FSL version 5.0.9). Subject-level models provided beta weight estimates for 1-back and 3-back conditions within each participant, relative to baseline fixation. All trial regressors were aligned to trial onset (duration = 1 s) and convolved with a double gamma hemodynamic response function. Because trials were closely spaced, the convolved 1-back and 3-back regressors approximated a block-based design, meaning that the observed PAG responses should be interpreted as an average across the 1-back/3-back block period (as opposed to more time-sensitive responses to the letter presented on each trial). Subject-level nuisance regressors included a run intercept, motion (i.e., translation/rotation in *x*/*y*/*z* planes), aCompCor components (five CSF, five WM; Muschelli et al., 2014), a discrete cosine transformation set with a minimum period of 264 s, and spike regressors (>0.5 mm framewise displacement; Satterthwaite et al., 2013). To assess multicollinearity, variance inflation factors (VIFs) were inspected for 1-back (VIF_mean _= 1.33; VIF_SD _= 0.16) and 3-back regressors (VIF_mean _= 1.48; VIF_SD _= 0.28), and were considered to be within an acceptable range (i.e., < 5; Mumford et al., 2015). Group-level analyses included whole-brain contrasts and analyses within PAG voxels (see below, PAG identification and alignment). Whole-brain contrasts computed *z*-scores relative to 0 for subject-level beta weights after smoothing with a 1 mm FWHM Gaussian kernel. Thresholds for *z-*score maps were corrected for multiple comparisons using threshold-free cluster-enhancement (TFCE; α < 0.05; Smith and Nichols, 2009).

### PAG identification and alignment

The PAG was identified and aligned across subjects using a semiautomated iterative procedure (for details, see Kragel et al., 2019), which built on manual PAG alignment methods previously used in 7 T studies of the PAG (for details, see Satpute et al., 2013). For each subject, a subject-specific mask of the cerebral aqueduct was created by identifying high-variance voxels in the region. Following this, subject-specific PAG masks were created by dilating the aqueduct mask (two voxels, i.e., 2.2 mm) and masking (1) voxels in the original subject-specific aqueduct mask, (2) voxels not in a subject-specific GM segmentation (>50% probability), and (3) any voxels outside the target range (MNI, −22 > *y* > −42; *z* > −14). This created a hollow, cylindrical PAG mask for each subject, which were aligned and warped to a group-average mask using DARTEL (differomorphic anatomical registration using exponentiated Lie algebra; Ashburner, 2007). Subject-group transformations were applied to whole-brain contrast images to align subject-level functional data to the common PAG-aligned space. For all analyses within the PAG, voxels were resampled to 0.5 mm isotropic space (from 1.1 mm isotropic native space), masked with the group-aligned PAG mask, and smoothed to 1 mm.

**Figure 2.**
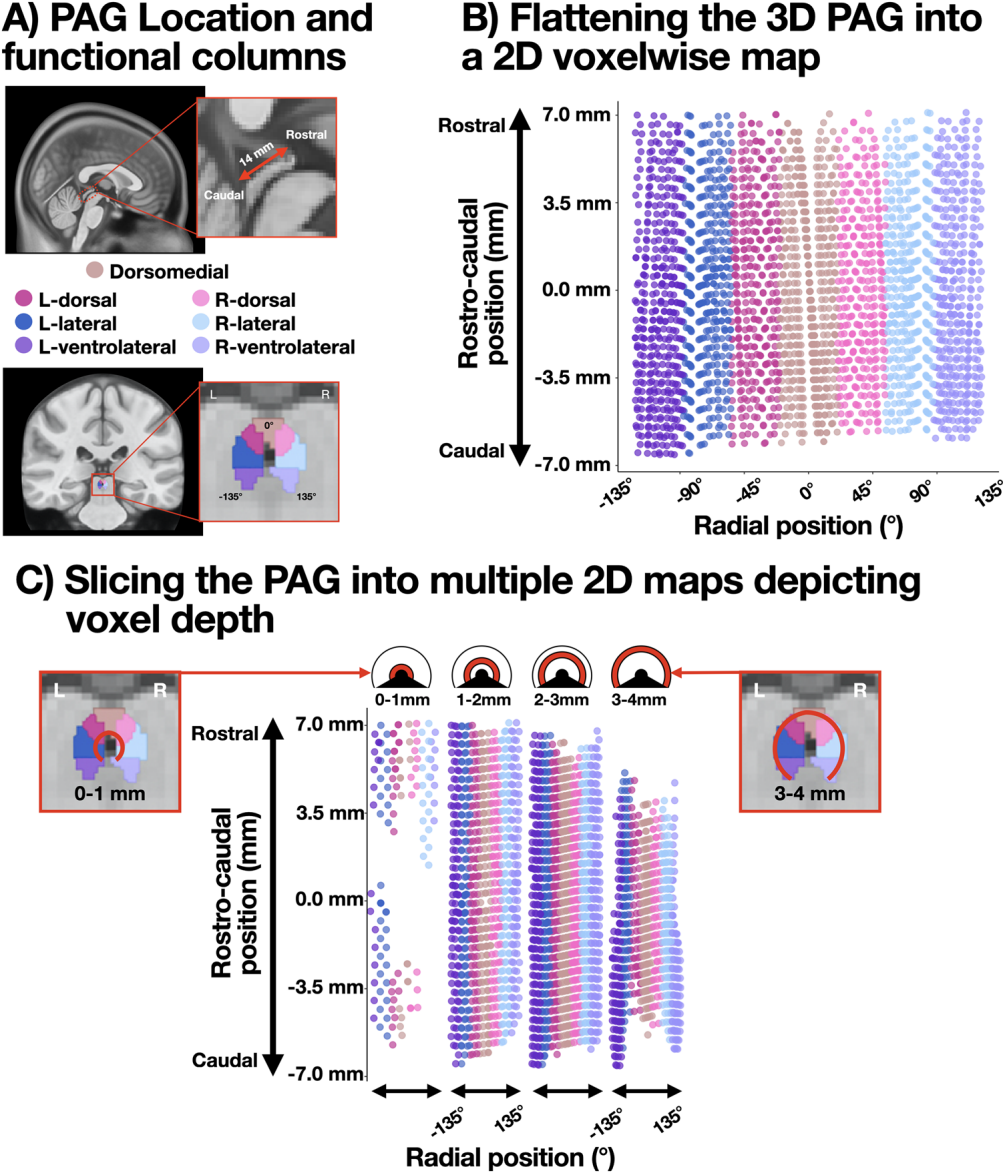
PAG identification, segmentation, and visualization. ***A***, Sagittal and coronal perspectives on the human PAG. For the purposes of visualization, functional columns within the PAG are colored according to the legend. The ventral 90° of the cylinder contains the dorsal raphe and was excluded from analysis (***B***) The PAG is a 3D structure and is difficult to visualize. To simplify the presentation of results, we labeled each voxel with its position on the rostral–caudal axis and its radial degree from the volumetric *y*-axis. Plotting the rostral–caudal position (*y*-axis) by the radial degree (*x*-axis) flattens the PAG into a 2D surface. This convention will be used in later figures. ***C***, The flattened map of PAG voxels can be simplified further by taking radial slices within the PAG. Each voxel was labeled with its radial distance from the center of the cylinder, and the radial distance is plotted panels depicting 1 mm bins. For example, the 0–1 mm panel shows voxels abutting the cerebral aqueduct, and the 3–4 mm panel shows voxels at the outer periphery of the PAG. As in (***B***), the *y*-axis illustrates the longitudinal (rostral–caudal) axis of the PAG. Within each bin, radial degrees are plotted on the *x*-axis.

Each voxel within the group-aligned PAG mask was tagged according to (1) its radial degree from the PAG midline, (2) its position along the rostral–caudal PAG axis, and (3) its radial distance from the center of the cylinder. Figure 2*B* depicts the “unrolled” PAG, with radial degrees on the *x*-axis, and rostral–caudal position on the *y*-axis. Figure 2*C* depicts the “unrolled” PAG “sliced” according to the radial distance from the center of the cylinder. The rostral–caudal position was calculated using the principal component analysis (PCA) on voxelwise *x*/*y*/*z* coordinates, and the radial degree and distance were calculated using a geometric transformation of those PCA coordinates, giving degree relative to the volumetric *y*-axis (at 0°) and cylinder center (at radial distance = 0). For visualization, Figure 2 depicts the functional columns within the PAG (Fig. 2); however, all group-level analyses were performed using either voxelwise data (Figs. 2, 5*A*) or bilateral ROIs aggregating across radial degrees or the rostral–caudal axis (Fig. 5*B*; see below, Linear mixed-effects model). The ventral most 90° of the PAG was excluded from analysis, given that it contains other subcortical structures (e.g., dorsal raphe).

### Linear mixed-effects model

Voxel beta weights within the PAG group-aligned mask were submitted to a linear mixed-effects model to summarize the spatial distribution of BOLD signal changes. PAG voxel beta weights were averaged within four ranks of the rostral–caudal position and 10 ranks of the radial degree (Fig. 5*B*). Each rostral–caudal rank consisted of a 3.5 mm segment along the 14 mm rostral–caudal positional axis, and each radial degree rank consisted of a bilateral average of 13.5° sections of the PAG—i.e., the first radial degree rank included 0–13.5° in the left and right PAG, while the last radial degree rank included 121.5–135° in the left and right PAG. Given this, our mixed-effects model assumes bilaterality in any effect along the PAG radial degree (an assumption we confirmed by visual inspection of the voxelwise pattern of results; Fig. 5*A*).

The mixed-effects model included fixed effects of condition (1-back/3-back), rostral–caudal rank, PAG degree rank, and all interactions. The model included by-participant random intercepts and by-participant random slopes for all fixed effects (Barr et al., 2013). Analyses were conducted in R (R version 3.6.2; R Core Team, 2016) using the lmer4 package (Bates et al., 2015), with degrees of freedom approximated by the Satterthwaite method, as implemented in the lmerTest package (Kuznetsova et al., 2017). The code was entered as follows:

$$\begin{array}{rl} & \mathrm{model}\;<-\;\mathrm{lmer}(\mathrm{averagedPAGBOLD}∼\mathrm{condition}\;*\\ & \;\mathrm{rostral}\text{–}\mathrm{caudal}\;\mathrm{position}\;*\;\mathrm{PAG}\mathrm{degree}\;+\;(\mathrm{condition}\;*\;\mathrm{rostral}\text{–}\mathrm{caudal}\\ & \;\mathrm{position}\;*\;\mathrm{PAG}\;\mathrm{degree}|\mathrm{participant}),\mathrm{control}\;=\;\mathrm{lmerControl}(\mathrm{optimizer} \end{array}$$

= *“bobyqa”, optCtrl* = list(maxfun = 2e5))).

### Peripheral physiological recording and quality assessment

Peripheral physiological measures were collected at 1 kHz using an ADInstruments PowerLab data acquisition system with MR-compatible sensors and LabChart software. Data were continuously acquired throughout the entire scan session and partitioned for alignment with fMRI data using experimenter annotations in LabChart and scanner TR events. A piezoelectric pulse transducer (PowerLab, ADInstruments) measured the heart rate from the left index fingertip. A respiratory belt with a piezoelectric transducer (UFI) measured RR and was placed around the chest at the lower end of the sternum.

Physiological time-series data were visually inspected for quality in Biopac Systems’ AcqKnowledge data acquisition and analysis software and in custom visualizations using R (R version 3.6.2; R Core Team, 2016) and the ggplot2 package (version 3.2.1; Wickham, 2009). Time-series were classified as high quality (i.e., few or no motion artifacts), noisy (i.e., IBI, motion artifacts affected <50% of each 1 min segment; RR, large, frequent motion artifacts or RRs outside of a typical range of 7–20 breaths per min), and unusable (i.e., IBI, containing excessive artifacts with few reliably detectable peaks; RR, containing low amplitudes that could not be distinguished from noise). Among the 87 participants with usable fMRI data, cardiac data was high quality (i.e., all 1 min segments were sufficiently free of motion to easily identify peaks in the IBI signal) for 59 participants, noisy for 12, unusable for 14, and missing for 2. Among the 87 participants with usable fMRI data, respiratory data was high quality for 47 participants, noisy for 20, unusable for 14, and missing for 6. Only high-quality data were used for cardiac (IBI) and respiratory (RR) analyses.

### Peripheral physiological preprocessing and analysis

Physiological time-series data were analyzed using MATLAB toolboxes and custom R scripts. The heart rate was calculated using the PhysIO MATLAB toolbox (Kasper et al., 2017), which used a 0.3–9 Hz bandpass filter, and identified peaks using a two-pass process. The first-pass estimated run average heart rate (inverse of IBI) detects peaks exceeding 40% of normalized amplitude, assuming a minimum peak spacing <90 beats per minute. Moreover, the first-pass peaks were used to create an averaged template, and the first-pass estimated average heart rate was used as a prior to detect peaks on the second-pass peaks (for more details, see Kasper et al., 2017). PhysIO pipeline-detected peaks were compared with peaks identified in Biopac by trained coders. Discrepancies between the two methods were rare (0.5% of peaks across all runs and participants) and occurred very rarely in the dataset scored by a more experienced coder (0.323% of peaks) compared with the dataset scored by a less experienced coder (0.878% of peaks). To reduce drift in the cardiac signal, heart rates were smoothed with a 6 s rolling average and down-sampled into scan TR. Upon completion of preprocessing, we then converted the heart rate into IBI, as IBI displays a more linear relationship with underlying autonomic cardiac inputs than the heart rate (Berntson et al., 1995). After converting HR (heart rate) to IBI, cardiac data were analyzed exclusively using IBI. RRs were calculated using custom R scripts. A 1 Hz low-pass filter was applied to the respiratory time-series, and local peaks were identified in a sliding 500 ms window. Small excursions (those <0.5 SD of average respiratory belt tension across the entire run) were deemed too small to meet criteria as a respiratory peak or trough. To maintain consistency between preprocessing of IBI and RR, RRs were also smoothed with a 6 s rolling average and down-sampled into scan TR. Down-sampling IBI and RR into scan TR resulted in a total of 263 captured instances of physiological activity for each variable (IBI, RR) in every participant; averages were then computed for each participant and for each resting fixation, 1-back and 3-back task epoch (see above, Experimental paradigm). Using the averaged task epoch values, change scores were computed as the difference in physiological activity (IBI, RR) between the resting fixation and the 1-back and 3-back task epochs.

## Results

### Performance and reaction time increased in the 1-back versus 3-back working memory task

As expected, and consistent with the earlier work using a small subset of this sample, performance (i.e., hit rates) was higher for 1-back trials (*M *= 0.99; *SD *= 0.02) than for 3-back trials [*M *= 0.89; *SD *= 0.08; *t*_(85) _= 12.18; *p *< 1e^−16^; *d *= 1.31]. Likewise, reaction times for correct responses were higher for 1-back trials (*M *= 0.76 s; *SD *= 0.16 s) than for 3-back trials [*M *= 0.99 s; *SD *= 0.22 s; *t*_(85) _= 13.75; *p *< 1e^−16^; *d *= 1.48]. Reaction times for incorrect responses did not significantly differ between 1-back and 3-back conditions.

### Peripheral physiology was affected by 1-back and 3-back working memory conditions

**Figure 3.**
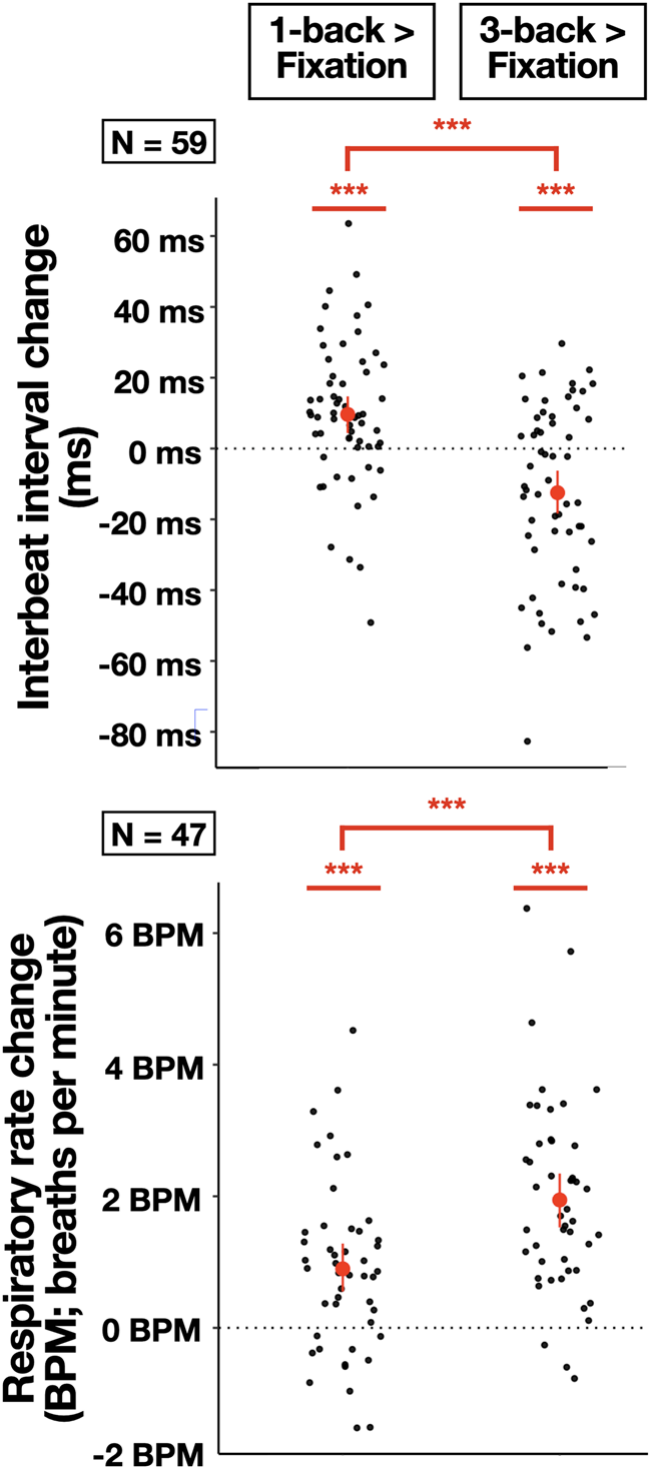
Task-dependent physiological changes from baseline. Group means and 95% confidence intervals are plotted in red for IBI and RR (respiration rate; see above, peripheral physiological recording and quality assessment). Black dots illustrate individual mean change across all 1-back (left) or 3-back (right) task periods. Note that a decrease in IBI reflects an increase in the heart rate. *** *p *< 0.001.

Group-level changes in peripheral physiology (IBI and RR) were compared with the resting baseline during 1-back and 3-back task epochs. Although previous analyses using a subset of the present sample did not observe any change in peripheral physiology from baseline (Kragel et al., 2019), we did observe changes in both 1-back and 3-back conditions (Fig. 3). In the 1-back condition, across participants, IBI increased (i.e., cardio-deceleration occurred), and reparatory rate increased compared with the resting baseline [IBI: *t*_(58) _= 3.64, *p *< 0.0006, *d *= 0.47; RR: *t*_(46) _= 4.75, *p *< 1e^−5^, *d *= 0.69]. In the 3-back condition, IBI decreased (i.e., cardio-acceleration occurred) and RR increased compared with the resting baseline [IBI: *t*_(58) _= 3.82, *p *< 0.0003, *d *= 0.50; RR: *t*_(46) _= 9.07, *p *< 1e^−12^, *d *= 1.32]. Relative to the 1-back condition, the 3-back IBI was shorter [*t*_(58) _= 5.21; *p *< 1e^−6^; *d *= 0.68], and the 3-back respiration rate was faster [*t*_(46) _= 6.07; *p *< 1e^−7^; *d *= 0.89]. Thus, both mildly and moderately difficult cognitive tasks elicited physiological changes: they both elicited increased RRs, and they elicited opposite cardiac responses, with the mildly difficult 1-back reducing the heart rate and the moderately difficult 3-back increasing it.

### Whole-brain contrast elicits expected pattern of cortical results and widespread brainstem activity in the 3-back condition

**Figure 4.**
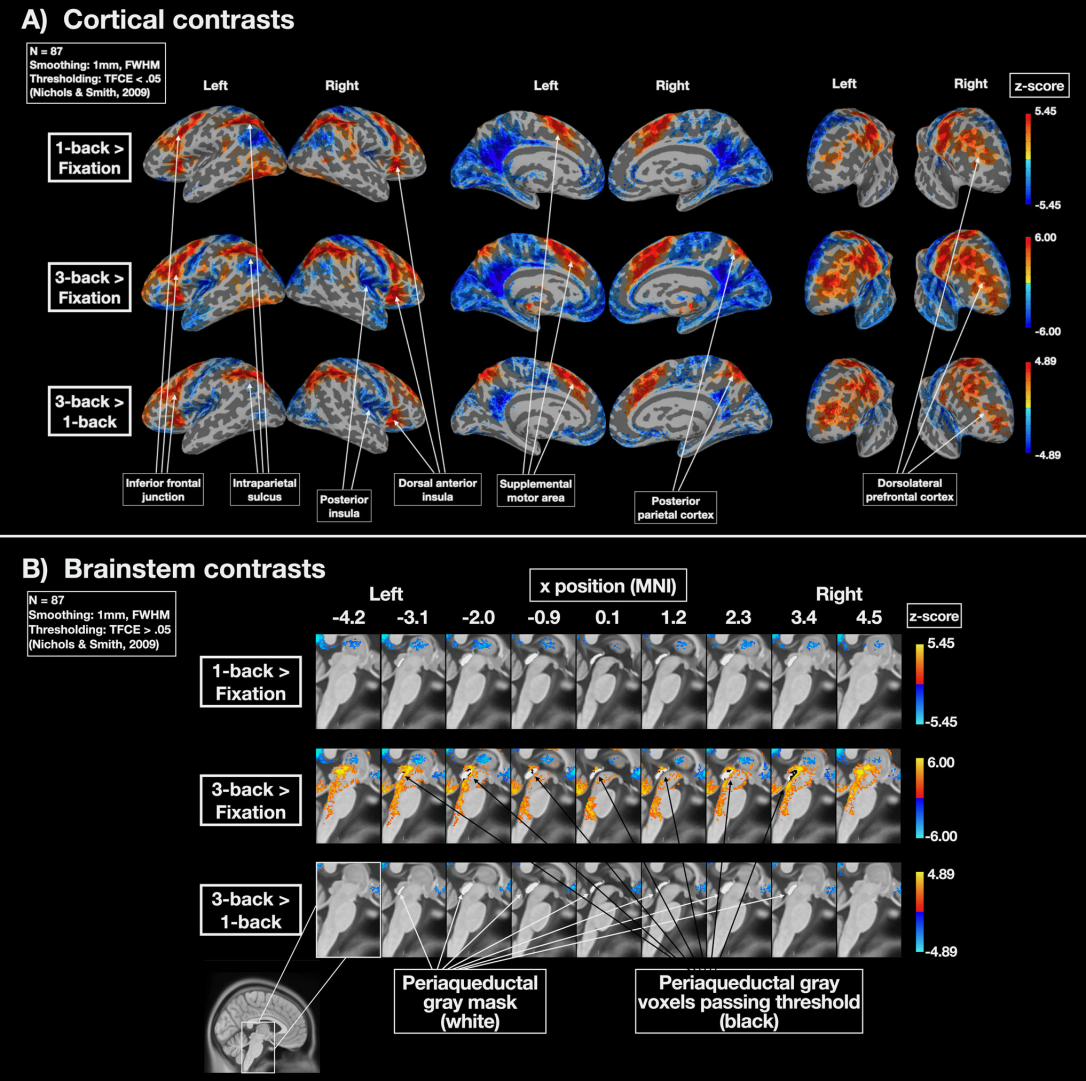
Group-level patterns in whole-brain and brainstem contrasts. ***A***, Whole-brain contrasts for 1-back > fixation, 3-back > fixation, and 3-back > 1-back contrasts. Subject-level estimates were smoothed with a 1 mm FWHM Gaussian kernel and submitted to a group-level GLM. Thresholding was performed using threshold-free cluster enhancement (α < 0.05; Smith and Nichols, 2009). The 1-back and 3-back > fixation contrasts generally showed a similar pattern within the cortex, with the exception in the posterior insula and posterior parietal cortex, where no 1-back response exceeding the TFCE threshold was observed. Cortical results for the 3-back > 1-back contrast were similar to those observed in prior work (Kragel et al., 2019), except that no 3-back > 1-back difference was observed in the PAG. ***B***, The 3-back > fixation contrast elicited widespread BOLD activity in the brainstem and in a number of voxels (marked in black) within the group-aligned PAG mask (marked in white). Notably, there was no 3-back > 1-back difference observed in the PAG or brainstem, suggesting that subtle changes above baseline were present in the 1-back condition as well. Voxelwise analyses within the PAG (Fig. 5) allowed us to examine this further. All subject-level contrasts were warped to a common PAG mask template to align PAG voxels at the group level (see Materials and Methods, PAG identification and alignment).

Whole-brain group-level analyses for 1-back, 3-back, and 3-back > 1-back contrasts were performed to ensure that cortical results were consistent with observations in prior work (Kragel et al., 2019). Group-level analyses were performed on first-level contrasts after applying a transformation to align the subject-specific PAG masks (see Materials and Methods, PAG identification and alignment), The 3-back > 1-back contrast was consistent with prior work, with bilateral responses observed in the inferior frontal junction, intraparietal sulcus, dorsal anterior insula, supplemental motor area, posterior parietal cortex, and dorsolateral prefrontal cortex (Fig. 4*A*). Notably, unlike in Kragel et al. (2019), we did not observe a suprathreshold response in the PAG for the 3-back > 1-back contrast. However, the 3-back > baseline contrast did elicit suprathreshold activity throughout the brainstem, including within the PAG (Fig. 4*B*).

The 1-back and 3-back (vs baseline) contrasts elicited similar patterns of BOLD response across the cortex (Fig. 4*A*), meaning that the 3-back > 1-back contrast estimates were generally caused by a difference in the intensity of the BOLD signal increase/decrease between the conditions. In other words, a similar set of brain regions responded to 1-back and 3-back conditions, but the magnitude of increase/decrease was greater for the 3-back condition (with the exceptions of the posterior parietal cortex and posterior insula, where no suprathreshold differences between the 1-back and resting baseline were observed; Fig. 4*A*). The fact that the 3-back condition elicited brainstem and PAG responses that exceeded baseline, but that did not differ from the 1-back response, suggested the presence of a subthreshold PAG response in the 1-back < baseline contrast as well. Given that the cortical response in 1-back and 3-back conditions were similar in their topographic organization, but different in their intensities, it was possible that a similar topographic similarity would be observed in the PAG as well. Extracting and analyzing the voxelwise topography of the BOLD response in the PAG allowed us to test this hypothesis.

### The voxelwise topography of the PAG BOLD response for 1-back and 3-back working memory conditions is strongly correlated

**Figure 5.**
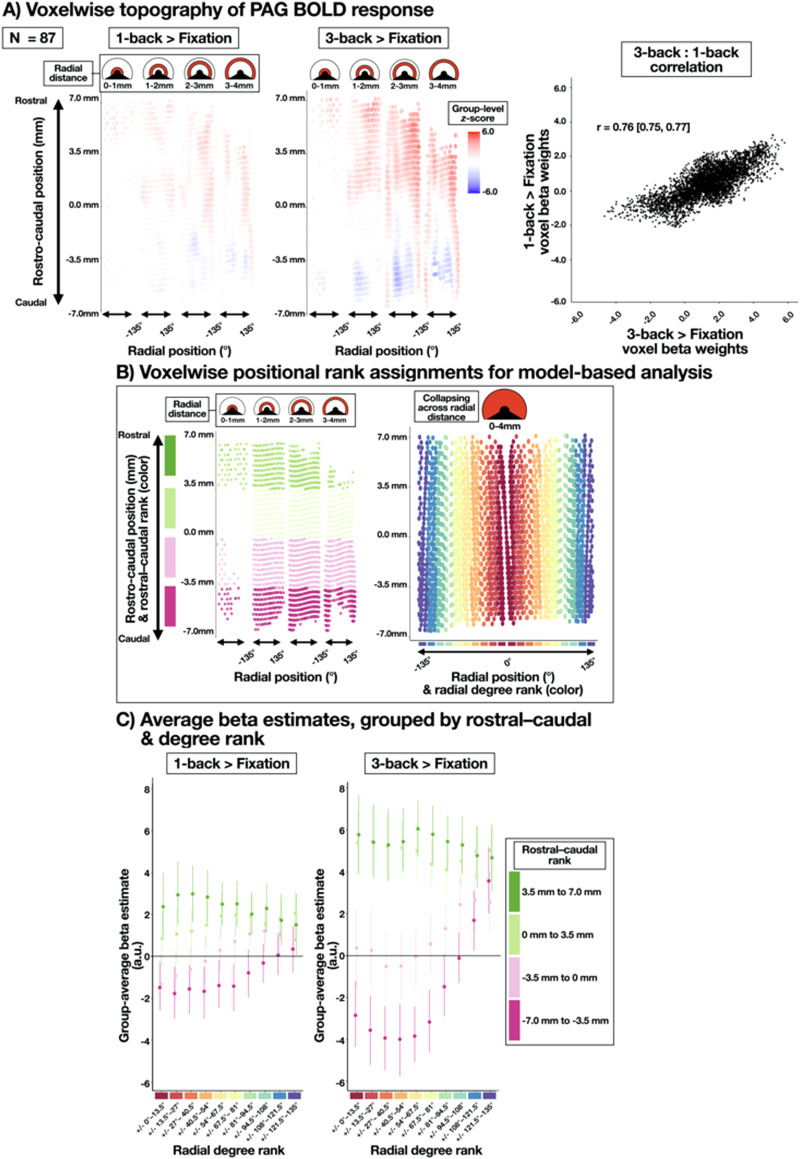
Patterns of group-level PAG BOLD signal intensity elicited by the 1-back/3-back task. ***A***, Left: Voxelwise topography of the PAG BOLD response (*z*-scored) during 1-back (left) and 3-back (right) conditions. Upper panels display radial slices of the PAG cylinder (each 1 mm thick; Fig. 2). The *y*-axis illustrates the voxel position along the rostral–caudal axis (14 mm in length). The *x*-axis illustrates the voxel position in ±135° from the brain midline. The heat map illustrates the group-level *z*-scored BOLD signal intensity relative to baseline fixation between task blocks. Voxels were interpolated to 0.5 mm isotropic, from 1.1 mm native space, and smoothed with a 1 mm FWHM Gaussian kernel. Right: Beta weights for all 1-back PAG voxels (*y*-axis) plotted against beta weights for all 3-back PAG voxels (*x*-axis). A Pearson’s correlation coefficient and 95% confidence interval were calculated using 10,000 bootstrapped samples. Across voxels, 1-back and 3-back *z*-scores were highly correlated, similar to 1-back and 3-back cortical responses, as reported in Fig. 4. ***B***, To model PAG topography in the 1-back and 3-back conditions, ordinal ranks were assigned to voxels according to their position on the rostral–caudal axis (4 ranks, left) and radial degree (10 ranks, right). Radial degree ranks were bilateral, meaning that our analysis assumed symmetry in any PAG response [which is visually supported by the topography in panel (*a*)]. In each subject, voxels in each combination of rostral–caudal and degree ranks were averaged to produce 40 estimates for each subject to be used in the mixed-effects model. ***C***, Subject-level beta weights were averaged and plotted (with 95% confidence intervals) for each combination of rostral–caudal and degree ranks. The *y*-axis, unlike in the plots above, depicts group-averaged beta weights (in arbitrary units, a.u.), and the *x*-axis depicts the radial degree rank (i.e., bilateral dorsomedial to ventrolateral regions, moving from left to right). In both 1-back (left) and 3-back (right) conditions, the rostral end of the PAG showed a general BOLD increase above fixation, whereas the caudal end of the PAG showed a dorsomedial decrease and increased BOLD response in ventrolateral areas. These plotted averages are consistent with the results of the mixed effects model (see Results).

Voxelwise *z*-scores for the 1-back and 3-back > baseline contrasts were plotted on a 2D surface to visualize the topography of the PAG BOLD response (Fig. 5*A*). Visual inspection of these results suggested that the 1-back and 3-back contrasts did elicit a PAG BOLD response that was spatially similar, but different in intensity, as in the cortical pattern of results. Supporting this topographic similarity, voxelwise *z*-scores were highly correlated between 1-back and 3-back contrasts; *r *= 0.76 (0.75, 0.77; bootstrapped 95% CI, 10,000 samples; Fig. 5*A*). To model the topographic pattern of the PAG response, we assigned two ordinal ranks to each PAG voxel: a rostral–caudal rank (four levels, from caudal to rostral poles of the PAG; Fig. 5*B*) and a radial degree rank (10 levels, from dorsomedial to ventrolateral PAG, averaging bilaterally; Fig. 5*B*; see Materials and Methods, Linear mixed-effects model). These ordinal ranks, along with the N-back condition (1-back, 3-back) and all interactions, were submitted to a linear mixed-effects model (including subject-level intercepts and all random slopes; Barr et al., 2013) to model the spatial topography of the PAG BOLD beta weights in both N-back conditions.

The main effect of the N-back condition was nonsignificant, [*F*_(1,86) _= 2.12; *p *< 0.15], consistent with the failure to observe any 3-back > 1-back difference within the PAG in the whole-brain contrast (Fig. 4*B*). The main effect of the rostral–caudal rank was significant, [*F*_(1,86) _= 52.61; *p *< 1e^−9^], but was qualified by an interaction with the N-back condition, [*F*_(1,86) _= 7.88; *p *< 0.007], suggesting that the linear increase along the rostral–caudal axis of the PAG occurred in both N-back conditions, but was greater in the 3-back condition than in the 1-back condition (Fig. 5*C*; *y*-axis increases from pink to green categories). The main effect of the degree rank was also significant, [*F*_(1,86) _= 41.92; *p *< 1e^−8^], and was also qualified by an interaction with the N-back condition [*F*_(1,86) _= 4.62; *p *< 0.035], suggesting that the linear increase from the dorsomedial to ventrolateral PAG occurred in both N-back conditions, but was greater in the 3-back condition than in the 1-back condition (Fig. 5*C*; *y*-axis increases from left to right along the *x*-axis). The magnitude of the linear increase from the dorsomedial to ventrolateral PAG also increased from the caudal to rostral end of the PAG (Fig. 5*C*), as indicated by an interaction between rostral–caudal rank and degree rank, [*F*_(1,86) _= 26.03; *p *< 1e^−5^], but no significant three-way interaction with the N-back condition was observed [*F*_(1,86) _= 3.64; *p *> 0.05], meaning that the spatial topography of the BOLD response in 1-back and 3-back conditions was similar along the dimensions of the rostral–caudal axis and radial degrees, and that the conditions did differ in the magnitude of change in the BOLD response along either spatial dimension of the PAG (Fig. 5*C*).

### Task-elicited changes in the peripheral physiological response correlate with PAG voxels, but the voxelwise topography of correlation is weakly related to task-elicited BOLD responses

**Figure 6.**
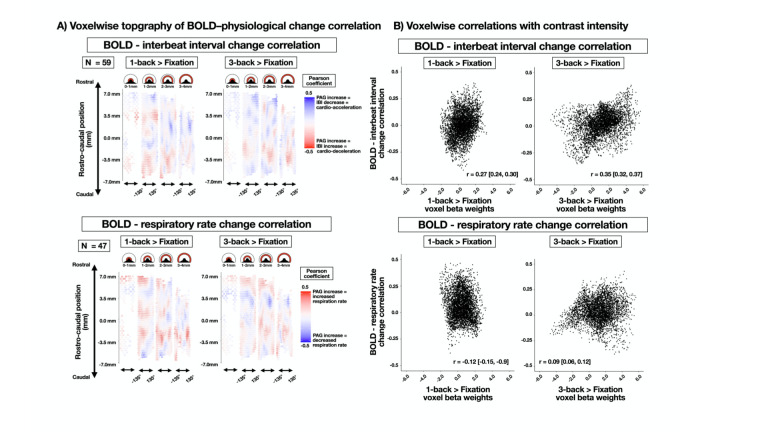
Voxelwise correlation with task-elicited physiological changes. ***A***, Voxelwise topography of between-subject correlations between either cardiac IBI (top) or RR (bottom) and estimated voxelwise PAG BOLD signal intensity (i.e., beta weight) elicited by the 1-back (left) and 3-back (right) task. Upper panels display radial slices of the PAG cylinder (each 1 mm thick; Fig. 2). The *y*-axis illustrates the voxel position along the rostral–caudal axis (14 mm in length). The *x*-axis illustrates the voxel position in ±135° from the brain midline. The heat map illustrates Pearson’s correlation coefficients. Correlations were calculated across all subjects with artifact-free physiological data (cardiac IBI = 69; RR = 47). ***B***, Voxelwise BOLD-physiological change correlation coefficients for IBI change (*y*-axis, top) and RR change (*y*-axis bottom) are plotted against contrast beta weights for all 1-back PAG voxels (*x*-axis, left) and 3-back PAG voxels (*x*-axis, right). Pearson’s correlation coefficients and 95% confidence intervals were calculated using 10,000 bootstrapped samples. Correlations across voxels were nonzero, but substantially lower than the correlation between the 1-back and 3-back voxelwise topography of BOLD response reported in Fig. 5.

The PAG is implicated in physiological regulation, and we expected that task-elicited changes in the PAG BOLD signal would correlate with changes (from baseline) in cardiac IBI and RR. To identify voxels that were associated with the magnitude of task-elicited physiological change from baseline, in each voxel, subject-level beta weights for 1-back and 3-back > baseline contrasts were correlated with subject-level changes in IBI and RR (Fig. 6*A*). The voxelwise spatial topography of the correlation coefficients was less clearly distinguishable along the expected organizational pattern of functional PAG columns than in the 1-back and 3-back > baseline contrasts (Fig. 5*A*). To directly compare these two patterns of voxelwise topography (i.e., voxelwise correlations with physiological change, Fig. 6*A*, and voxelwise task-elicited BOLD increases/decreases, Fig. 5*A*), we plotted the estimates against each other (Fig. 6*B*). We observed moderate correlations across PAG voxels between task-elicited BOLD and its correlation with changes in cardiac IBI (Fig. 6*B*, top), but weaker or negative correlations with change in RR (Fig. 6*B*, bottom). In all cases, the similarity in topography was much weaker than the similarity between the PAG BOLD signals elicited in the 1-back and 3-back tasks.

## Discussion

Our results suggest that, in humans, nonpainful and nonthreatening tasks that elicit mild physiological changes elicit a spatially organized pattern of BOLD response in the PAG. Further, the moderate and mild working memory demands of the 3-back and 1-back tasks elicited nearly identical patterns of BOLD response across the PAG—the 3-back response magnified the intensity of response, but did not change its voxelwise topography. In both 1-back and 3-back conditions, BOLD increases were observed along the entire rostral–caudal axis in the vlPAG, suggesting that the BOLD response to the cognitive task is organized along the previously known anatomical boundaries of PAG functional columns (for review, see Carrive and Morgan, 2012).

It was surprising that light cognitive exertion would elicit a localized BOLD response in the vlPAG, as previous studies have associated the function of the vlPAG with survival-related stimuli, including stimuli eliciting “freezing” behavior (in rats; Carrive et al., 1997; Morgan et al., 1998), and anticipated breathlessness or subcutaneous pain in humans (Faull et al., 2016; Tinoco Mendoza et al., 2023). Given that the present work observed a clearly defined pattern of BOLD increase in the vlPAG in response to a task approximately as easy as remembering a phone number, we posit that the PAG plays a more general functional role than just supporting survival-related behavior (e.g., housing “fear” or “survival” circuits; Panksepp, 2011; Silva et al., 2016; LeDoux and Daw, 2018; McNaughton and Corr, 2018; Lu et al., 2022; Vázquez-León et al., 2023)—consistent with accumulating evidence that the PAG helps coordinate and regulate the internal (visceromotor) systems of the body (for similar perspectives, see Benarroch, 1993, 2001; Faull et al., 2019). In other words, although prior work has observed associations between PAG activity and “fight-or-flight” or “freezing” behavior in the context of threat, the overall function of the PAG may be better described by its support for autonomic regulation in many situations, even in contexts where survival-related threat is absent, such as the working memory context used in the present work.

Within the past decade, significant progress has been made in imaging the human PAG (Coker-Appiah et al., 2013; Hermans et al., 2013; Lindner et al., 2015; Weis et al., 2020; Zhou et al., 2021; Wang et al., 2022; Weis et al., 2022; Tinoco Mendoza et al., 2023); however, because the PAG has been associated with survival-related behavior, most experiments continue to explore its function in these threat-based contexts. In contrast, the N-back task used in the present work involves cognitive demands that are within the range of what is experienced in daily life and provides a context in which to probe the PAG BOLD response during less affectively evocative task conditions. Successful performance in a N-back working memory task requires integrating exteroceptive (e.g., visual stimuli) and interoceptive sensory signals (e.g., signals of energy status from the body), maintaining those signals across trials (i.e., working memory), mobilizing energy to meet changing task demands (e.g., as reflected in changes in cardiorespiratory reactivity; Thayer et al., 2012; Dampney et al., 2013; Berntson et al., 2017; Hoemann et al., 2020), maintaining goal-directed behavior (e.g., allocating attentional resources), and finally, generating and executing a motor plan (e.g., pushing a button to make a response). These brain functions support successful working memory performance, but they also support other complex, goal-directed sensorimotor behaviors. Indeed, these are the basic operations that are needed to perform many laboratory psychological tasks and engage in real-world motivated performance (i.e., situations that are goal-relevant to the performer, require instrumental cognitive responses, and are active rather than passive; Blascovich and Mendes, 2000). From this perspective, the working memory task is a motivated performance task, similar to other motivated performance tasks that have elicited a PAG BOLD response under more stressful conditions (e.g., social stress during preparation for public speaking; Wager et al., 2009a,b; Theriault et al., under review). The N-back task, then, in addition to being regarded as a prototypical “cognitive” task, can also be thought of as a task that drives the brain to mobilize resources to meet task demands—i.e., to engage in allostatic regulation (Barrett, 2017; Sennesh et al., 2022). The PAG is anatomically well positioned to contribute to this allostatic function—especially given the its anatomical position at the convergence of ascending viscerosensory signals from the body and descending visceromotor signals for controlling the body (for review, see Carrive and Morgan, 2012).

In sum, our observation that both mildly and moderately challenging cognitive tasks elicited similar patterns of BOLD response in the PAG is consistent with the emerging perspective that the PAG serves a general integrative function—as opposed to a survival-specific function—and may support the integration of bottom-up sensory signals from the body with top-down visceromotor control signals from the brain (for review, see Bandler et al., 2000; Benarroch, 2012; Gianaros and Wager, 2015; Koutsikou et al., 2017). This perspective was hindered by the rarity of studies probing PAG function outside of threat-related contexts, and so future work would benefit from exploring PAG function in naturalistic conditions, and in other experimental contexts, such as the N-back task, that drive the brain to mobilize resources for action. Such studies could identify subcortical responses that might be missed in experimental contexts that exclusively study rare and intense events that trigger a “fight-or-flight” response. Indeed, the fact that moderate cognitive demand elicited a BOLD increase across the entire brainstem (including the PAG; Fig. 4*B*) suggests that there is a great deal to learn about brainstem and subcortical contributions to cognition.
